# FACTORS AFFECTING THE ACCEPTANCE OF CARE ROBOTS BY OLDER ADULTS: FOCUSING ON TECHNICAL FACTORS

**DOI:** 10.1093/geroni/igad104.3784

**Published:** 2023-12-21

**Authors:** Hee Jeong Yoon, Hyun Ju Lee, Si Woo Ban, Ha Yul Kim, Eun Gyo Cha, Jung Wan Lee, Hye Ri Shin, Young Sun Kim

**Affiliations:** Kyung Hee University, Seoul, Republic of Korea; Kyung Hee University, Seoul, Republic of Korea; Kyung Hee University, Seoul, Republic of Korea; Kyung Hee University, Seoul, Republic of Korea; Kyung Hee University, Seoul, Republic of Korea; Kyung Hee University, Seoul, Republic of Korea; Kyung Hee University, Seoul, Republic of Korea; Kyung Hee University, Seoul, Republic of Korea

## Abstract

Care robots have the potential to support the healthy and independent lives of older adults and alleviate the burden on care workers. However, the potential benefits of care robots can only be realized if they are accepted. Given that most older adults do not have experience with care robots and lack digital literacy, an understanding of the factors affecting the acceptance of care robots is important. The goal of this study was to analyze the factors that affect older adults’ acceptance of care robots by distinguishing the factors into sociodemographic, health, and technical factors. Data were collected from older adults aged 60 or over living in the community of Korea in 2022 (N= 506, mean age=70.11±7.74, women=54.55%). Hierarchical regression analysis was conducted to identify the factors related to acceptance of care robots. The results showed that factors influencing older adults’ acceptance of care robots were significant in sociodemographic factors (education and place of residence), health factors (self-reported health and instrumental activities for daily living), and technical factors (digital literacy and technostress). The findings indicate that interventions are needed to increase the acceptance of care robots by vulnerable groups with low levels of education, living in rural areas, and poor health conditions, and that it is also important to increase their digital literacy and lower their technostress. This study would provide valuable guidelines to stakeholders such as service providers, and policymakers to increase the acceptance of care robots and improve the quality of life of older adults.

